# Availability and Cost of Healthy Foods in a Large American Indian Community in the North-Central United States

**DOI:** 10.5888/pcd15.170302

**Published:** 2018-01-04

**Authors:** Amanda M. Fretts, Corrine Huber, Lyle G. Best, Marcia O’Leary, Laurel LeBeau, Barbara V. Howard, David S. Siscovick, Shirley A. Beresford

**Affiliations:** 1 Department of Epidemiology, University of Washington, Seattle, Washington; 2 Missouri Breaks Industries Research Inc, Eagle Butte, South Dakota; 3 MedStar Health Research Institute and Georgetown and Howard Universities Center for Translational Sciences, Washington, District of Columbia; 4 New York Academy of Medicine, New York, New York

## Abstract

To understand the local food environment in a rural American Indian community, we assessed the availability and price of healthy foods offered at all stores (n = 27) within a 90-mile radius of the town center of a large American Indian reservation. Stores were classified by type, and availability and cost of foods were measured using the Nutrition Environment Measures Survey in Stores (January–February 2016). Healthy foods were available at most grocery stores (>97%), although the price of foods varied considerably among stores. Having quantified the availability and cost of food, efforts must focus on understanding other structural and contextual factors that influence diet in this community.

## Introduction

Social, economic, and geographic factors influence diet and food-purchasing patterns, including the low availability and high cost of foods (1–5). The purpose of this study was to understand the food environment in a large American Indian community in the north-central United States. The community is classified as a food desert by the US Department of Agriculture (USDA) Economic Research Service, with 37% to 72% of residents (census tract dependent) living 10 or more miles from a grocery store (6). Understanding the availability and cost of foods in this community can inform development of culturally appropriate and community-targeted healthy diet programs.

## Methods

The availability and cost of 68 food items that comprise the Nutrition Environment Measures Survey in Stores (NEMS-S) were assessed at all retail food stores in a large American Indian community in the north-central United States, in January and February 2016. Surveyed stores included all businesses that sold food within a 90-mile radius of the town center of the reservation and included stores on and off the reservation. This is a rural area with a high level of poverty (>25% of families below the federal poverty guidelines) (7). The institutional review board of the University of Washington, the Indian Health Services, and the tribal health board approved the study, and all stores were approached individually to solicit participation.

NEMS-S is a commonly used instrument to evaluate the food environment and comprises food items needed to adhere to the USDA Thrifty Food Plan (TFP). The TFP market basket represents the minimal cost of a healthy diet for a family of 4 for 1 week (8). A trained member of the study staff (L.L.) collected the price and availability of the 68 food items that NEMS-S comprises at all stores through store visits (8).

Two study staff members reviewed the completed NEMS-S and double-entered data into Research Electronic Data Capture, version 7.6 (Vanderbilt University), to minimize data entry errors. The data were exported to Stata, version 13.0 (Stata Corp), for analyses. Stores were classified by type (convenience, dollar/discount, grocery, or supermarket), and analyses were performed in aggregate. Availability as well as mean and median cost of the food items and the TFP market basket were assessed by using the standardized procedures outlined in the NEMS-S manual (8).

## Results

Thirty businesses that sold food within 90 miles of the reservation’s town center were identified. NEMS-S was completed at 27 stores (3 stores declined to participate). Cities and towns with 1,000 or more residents had 73% of the grocery stores and the discount supermarket, 100% of discount/dollar stores, and 62% of convenience stores. Of all surveyed stores, 10 were on the reservation, including 4 grocery stores. All foods were available at the discount supermarket, and on average, 97% of foods were available at the grocery stores (Table 1). Convenience and discount/dollar stores were less likely to carry the foods, stocking on average 6% (convenience stores) and 36% (discount/dollar stores) of NEMS-S foods.

**Table 1:** Availability of Foods, by Store Type, January–February 2016^a^

Food^b^	Convenience Store, N = 13		Discount/Dollar Store, N = 3		Grocery Store, N = 10		Discount Supermarket, N = 1
	Median No. Foods Available, (%)	Range	Median No. Foods Available, (%)	Range	Median No. Foods Available, (%)	Range	Foods Available, (%)
Fresh fruits and vegetables (12)	0	0–1	0	0–0	11 (92)	10–12	12 (100)
Canned or frozen fruits and vegetables (10)	0	0–5	5 (50)	4–6	10 (100)	10–10	10 (100)
Breads, cereals, and grains (15)	0	0–8	7 (47)	4–8	15 (100)	13–15	15 (100)
Dairy (6)	2 (33)	0–5	5 (83)	1–6	6 (100)	5–6	6 (100)
Fresh meat and meat alternatives (7)	0	0–5	3 (43)	0–4	6 (86)	4–7	7 (100)
Frozen or canned meat and meat alternatives (5)	0	0–3	0	0–2	5 (100)	4–5	5 (100)
Fats, oils, sugar, and sweets (13)	1 (8)	0–11	6 (46)	5–8	13 (100)	4–4	13 (100)

a Assessed by using the Nutrition Environment Measures Survey in Stores in a rural American Indian community in the north-central United States.

b Numbers in parentheses are percentages of foods available in each category.

The cost of the TFP was 15% lower at the discount supermarket than at grocery stores ($152.91 at the discount supermarket vs mean $179.52 [range: $146.32–$199.98] at grocery stores) (Table 2). However, the cost of foods that made up the TFP market basket varied across food groups. For instance, the mean cost of dairy products was 43% lower at the discount supermarket than at the grocery stores, while the cost of fresh fruits and vegetables was 6% higher at the discount supermarket than at the grocery stores (Table 2). Convenience and discount/dollar stores did not carry enough of the food items that NEMS-S comprises to fulfill the TFP; 94% (range: 46%–100%)of foods were unavailable at convenience stores and 64% (range: 60%–71%) were unavailable at discount/dollar stores. Neither the availability of the foods that NEMS-S comprises nor the cost of the TFP market basket differed when analyses were stratified by location (on reservation vs off reservation) or when restricted to stores within a 50-mile radius of the reservation’s town center.

**Table 2:** Price of Foods, by Store Type, January–February 2016^a^

Food	Grocery, N = 10		Discount Supermarket, N = 1
	Price, Mean $	Range, %	Price, $
Fresh fruits and vegetables	44.26	25.12–67.29	46.95
Canned or frozen fruit and vegetables	25.31	19.21–30.94	16.38
Breads, cereals, and grains	22.65	18.67–25.77	17.94
Dairy	15.51	12.61–21.17	8.87
Fresh meat and meat alternatives	46.81	36.52–69.12	41.06
Frozen or canned meat and meat alternatives	15.73	13.60–18.26	14.82
Fats, oils, sugar, and sweets	9.25	7.69–11.84	6.89
Thrifty Food Plan, $^b^	179.52	146.32–199.98	152.91

a Assessed by using the Nutrition Environment Measures Survey in Stores in a rural American Indian community in the north-central United States.

b Nationwide, the average price of purchasing all foods that the Thrifty Food Plan comprises was $151.20.

## Discussion

The foods that NEMS-S comprises were largely available at grocery stores and at the supermarket in the rural American Indian community we studied, although the price of purchasing the foods varied considerably by store. During the time that NEMS-S was administered in the community, USDA estimated that the average cost to purchase a TFP was $151.20 (9). Thus, the cost of purchasing a TFP market basket ranged from 3% lower to 24% higher than the national average in this American Indian community. This is similar to results found in other studies, which demonstrated substantial variation in the cost and availability of healthy foods (1,4,10,11).

This study has strengths and weaknesses. This study was an ancillary project of the Strong Heart Study, and we worked through that study’s established channels to maximize the project’s efficiency. All stores were surveyed only once, and we were unable to account for variation in availability and price of foods over time. Although we attempted to survey all stores that sell food within the 90-mile catchment area, 3 convenience stores refused to participate. Finally, the study was designed to better understand the availability and cost of foods, and we did not evaluate other structural barriers (eg, transportation, food quality, travel distance) that may affect a person’s ability to consume a healthy diet, nor did we evaluate individual purchasing behaviors.

This study indicates that healthy food options were available in the community studied, although the price of these foods appeared to be slightly higher than the national averages and varied considerably across stores. Greater efforts are needed to better understand a wider range of contextual and structural factors that influence food-purchasing patterns beyond availability and cost.
